# PET/MR: primary inferior vena cava leiomyosarcoma

**DOI:** 10.1186/s41824-022-00144-3

**Published:** 2022-11-01

**Authors:** Brunela Ronchi, Gustavo Agustin Peña, Carlos Sacchi

**Affiliations:** Department of Nuclear Medicine, Foundation School of Nuclear Medicine (FUESMEN), Mendoza, Argentina

**Keywords:** PET/MR, Leiomyosarcoma, Retroperitoneal tumor, FDG, Vascular tumor, Vena cava

## Abstract

Positron emission tomography (PET) combined with a magnetic resonance (MR) scanner (PET/MR) with 18F-fluorodeoxyglucose (FDG) tracer is being used in quite a few nuclear medicine centers. The aim of this study is to illustrate two uncommon cases of primary inferior vena cava leiomyosarcoma which were formerly evaluated with anatomical images such as computed tomography and ultrasound. These techniques were inferior in the definition of the tumor and its characteristics. F-18 FDG PET/MR was essential and provided all the necessary information: its origin, local extension, anatomo-metabolic behavior, form of presentation, and distant metastasis in one single diagnostic technique. PET/MR accurately contributed to the diagnosis in a shortened period of time and, therefore, in the prognosis of this disease with greater benefits.

## Introduction

Inferior cava vein leiomyosarcomas (LMS) are an unusual entity, rising from smooth muscle cells with low growth rates. These features show the low recognition of this pathology, mainly due to its infrequency. Our medical staff was motivated to perform more complex studies available in our region, in order to improve localization accuracy of the suspected neoplasm, proving a precise diagnosis of an uncommon disease, later confirmed by histological studies.

Positron emission tomography (PET) combined with a magnetic resonance (MR) scanner (PET/MR) imaging plays a pivotal role in the abdominal evaluation and characterization of retroperitoneal tumor lesions and extension to neighboring organs. The shortage of literature and case reports promoted a bibliography discussion and the presentation of the cases.

## Case presentation

The first patient, a 50-year-old woman, starts with prolonged abdominal pain and distension. An abdominal ultrasound is performed which shows an expansive hypoechoic, heterogeneous mass in the retroperitoneum. Consequently, a non-contrast computed tomography (CT) was performed to characterize the lesion. It exhibited a hypodense mass which displaced neighboring organs with no clear diagnosis.

**Fig. 1:**
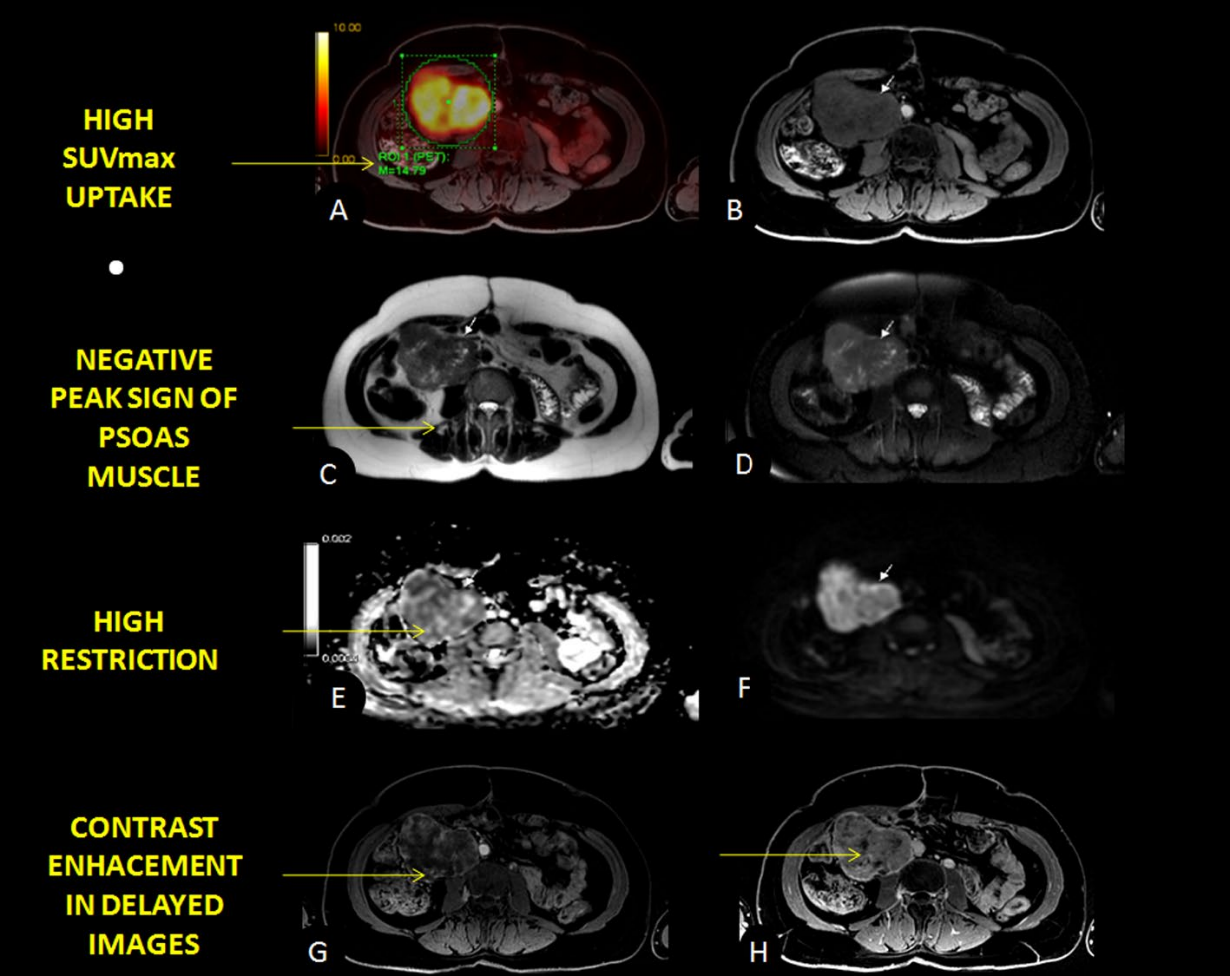
PET/MRI (**A**) images illustrate solid and hypermetabolic (elevated FDG uptake SUVmax 11.91) expansive formation in the retroperitoneum. It can be seen as iso-/hypointense on T1 (**B**) and heterointense on T2 and T2 FAT SAT (**C**, **D** respectively), with well-defined and lobulated borders. It presents a heterogeneous enhancement after IV contrast (**G**), due to necrosis areas, high restriction on diffusion images (**E**–**F**) and contrast enhancement intensifies in delayed images (**H**). Negative peak sign from psoas muscle was useful in the diagnosis (see description below) (**B**–**D**)

A retroperitoneal neoplasm is suspected, specifically a sarcoma; thus, since our center has a PET/MR, it was executed (Fig. 1). PET/MR shows a retroperitoneal hypermetabolic (SUVmax 11.91) lobulated, nodular image of 8 × 5 cm, that compresses vascular structures and depends on them. It also displaces other organs such as the right kidney and lower portion of the liver. IV contrast enhancement intensifies in late images.

**Fig. 2:**
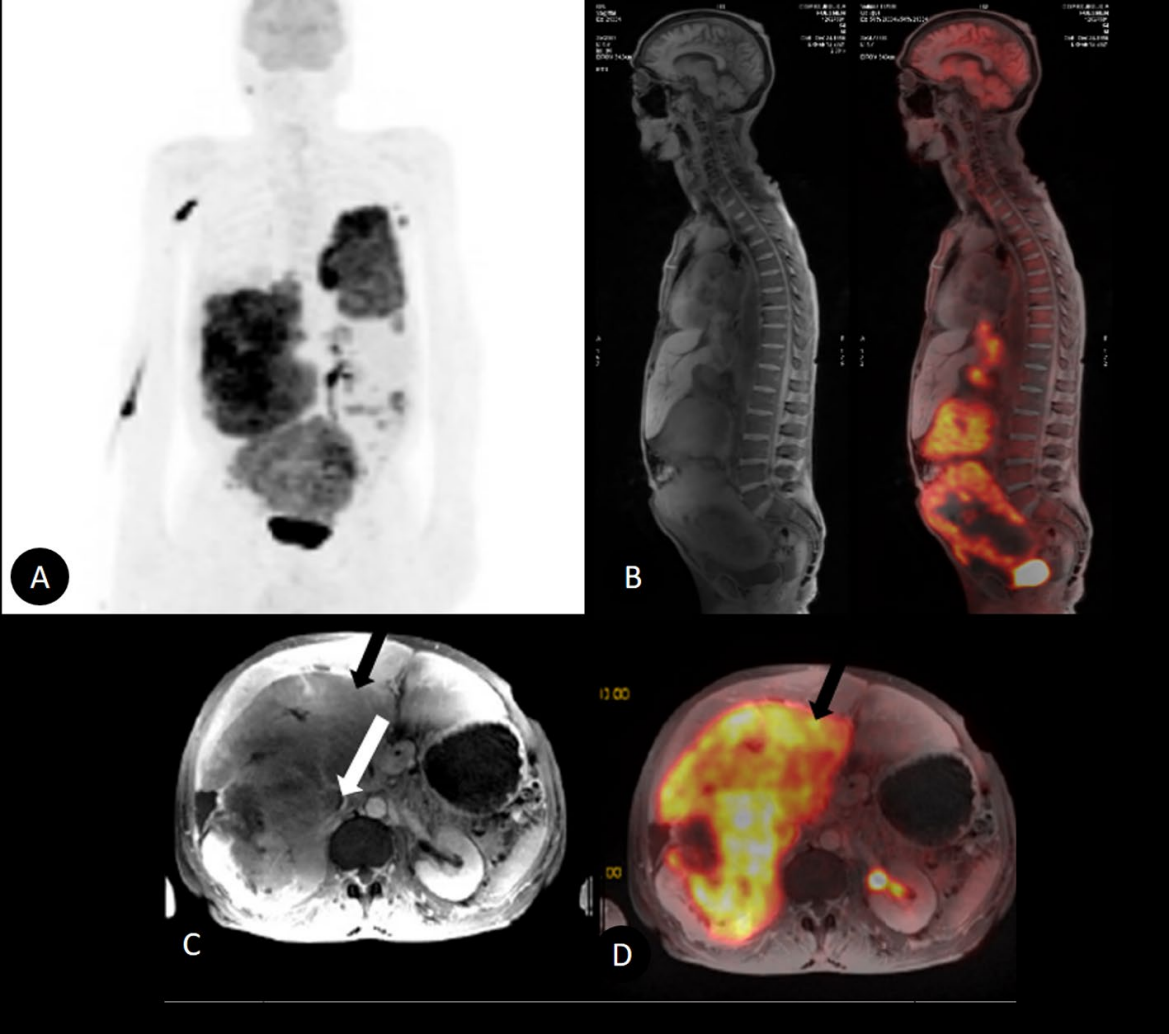
MIP images **A** showed a hypermetabolic (black arrow: SUV max 13.95) voluminous expansive mass in the retroperitoneum and abdominal cavity. PET/MRI sagittal fusion images **B** illustrate the extension and compromise of distant organs. PET/MRI axial images **C**, **D** demonstrate the origin of the tumor from IVC (white arrow) with an eccentric growth

Our second patient is a 64-year-old male with a history of drug addiction, weight loss and abdominal pain. Firstly, a CT scan showed moderate left pleural effusion, with a voluminous expansive lesion of 12.3 cm in contact with the upper pole of the right kidney, and with compressive effects on the liver parenchyma. After a suitable evaluation in which renal or adrenal origin was considered, a PET/MR scan was necessary to distinguish the tumor properly and its extension (Fig. 2).

For these complex cases, PET/MR was paramount to previously identify the site of origin of the neoplasm, extension, metabolic grade and distant metastasis and subsequently provide correct treatment.

Both patients were evaluated with PET/MR with F-18 fluorodeoxyglucose (FDG), in a full-body PET 3D TOF acquisition scan in Hybrid Resonator, PET/MR General Electric SIGNA 3 Tesla. Pathological studies with immunostaining showed high-grade spindle cell sarcoma in both patients. Consequently, they both commence chemotherapy treatment. The former patient additionally underwent radiation treatment with positive outcomes with regular follow-up imaging to monitor for recurrences. Sadly, the second patient died with no further image evaluation.

## Discussion

Inferior cava vein (IVC) leiomyosarcomas (LMS) are an unusual entity, rising from smooth muscle cells of the media, although it is the most frequent primary tumor from IVC. In general, LMS of the IVC account for 5% of all vascular LMS. Vascular LMS tumors represent about 1–2% of all LMS, which accounts for 10–20% of all sarcomas in correlation with the low proportion of cases described in the literature. Sarcoma represents 1% of all adult malignancies (Sephien et al. 2019). Epidemiologically, LMS have been reported in women in a slightly higher proportion around 50 to 60 years old (Reddy et al. 2010). Poor prognosis has been described among the scarce literature found (Sephien et al. 2019; Ghose et al. 2018) and a life expectancy of 5 years (Punt et al. 2009).

**Fig. 3:**
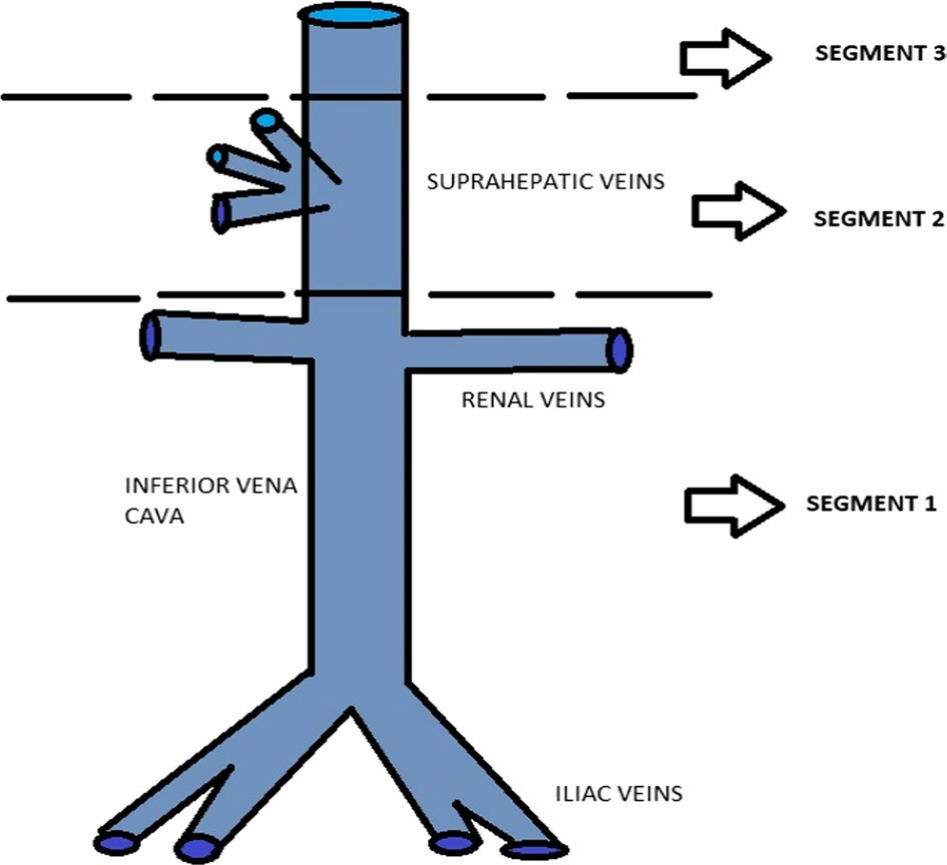
Mingoli’s anatomical classification of IVC LMS

IVC leiomyosarcomas are retroperitoneal neoplasms with different growing patterns: extraluminal (major proportion), intraluminal or mixed patterns. They could also be divided into an anatomical classification formerly described by Mingoli (Fig. 3). The first segment includes all venous structures under the renal veins, the second segment is described between the renal veins and the suprahepatic veins, and the third one from the suprahepatic veins to above, into the right atrium (Sephien et al. 2019). LMS affects them in 35%, 45% and 20%, respectively, although the whole cava vein may be involved in around 10–17% of the cases described (Ghose et al. 2018; Monteagudo et al. 2015; Zhou et al. 2020). Considering our patients, we may report that patients number 1 and 2 are anatomically classified as segments 1 and 3, respectively. Further, as they show a slow growth rate, LMS are usually detected as large, lobulated, irregular and heterogeneous masses. Few studies have notified an average length of 10 cm, reporting measurements between 5 and 20 cm. (Punt et al. 2009; Webb et al. 2013; Zhou et al. 2020; Blum et al. 1995). The size of the tumor in the first and second patient was 8 cm and 12 cm, respectively.

The slow rate of sarcomas and the type of growth reflect the variety of symptoms and the late clinical presentation: from asymptomatic to vague abdominal pain, distension, palpable mass, obstructive syndrome, Budd–Chiari syndrome, edema, hypertension and tumor thrombosis (Bednarova et al. 2018). Despite its low metastatic potential, the most common sites are liver, lungs, lymph nodes and bone through vascular and lymphatic dissemination (Sephien et al. 2019; Reddy et al. 2010; Ghose et al. 2018; Bednarova et al. 2018), also into the abdominal aorta, right kidney, adrenal and colon due to its nearness (Sephien et al. 2019; Reddy et al. 2010; Ghose et al. 2018). Most important characteristics of LMS are summarized in Table 1.

**Table 1:** Inferior vena cava leiomyosarcoma key features

Inferior vena cava leiomyosarcoma key features
Most frequent primary tumor from IVC
Middle-age women
Lobulated, irregular and heterogeneous mass
Extraluminal growth
Average length of 10 cm
Most common location between the renal veins and the suprahepatic veins
Low metastatic potential

The differential diagnoses include the wide spectrum of retroperitoneal mass that could be divided into two groups. In the pediatric population, adrenal neuroblastoma and renal nephroblastoma are the top two in prevalence (Sephien et al. 2019). Cholangiocarcinoma, duodenal malignancies, angiosarcoma, neurogenic tumors, retroperitoneal fibrosis, retroperitoneal lymphoma and adrenal pheochromocytoma, renal cell carcinoma and pseudolipoma need to be contemplate among adults due to their similar imaging characteristics such as enhancing solid mass, necrosis and hemorrhagic (Sephien et al. 2019; Molina et al. 2016).

It is admitted that histological grade is the most important prognostic factor for adult soft tissue sarcomas. Consequently, the proficiency of PET/MR images to provide a metabolic grade is of utmost importance in the prognosis of these patients, as it may differentiate low grade from high-grade sarcomas in an imaging scan with considerable anticipation to biopsy results. Sarcomas are a complex and non-homogeneous group of neoplasms in their anatomical and histological presentations. According to an extended bibliography revision, the most commonly used are the French grading and the National Cancer Institute grading systems. Both of them have 3 grades and are based on mitotic activity, necrosis and tumor differentiation. The practical value of these scores relies on the type of treatment each tumor is sensible too. In this way, some of them are more chemotherapy or radiotherapy sensible, concluding that the histopathologic subtype is inextricably linked with the prognosis of the patient (Coindre et al. 2006).

### Imaging

18F-FDG PET/MR owns the advantages of both separate methods combined in one single diagnostic approach. The three-dimensional capability of MR imaging allowed it to delimit the vascular and neural structures with more accuracy, under considerably less exposure to radiation. MR also has a higher soft tissue resolution, with accurate visualization during the portal phase (Bednarova et al. 2018). Leiomyosarcomas are common hypovascular structures; however, they express peripheral enhancement through this imaging modality (Ghose et al. 2018). LMS are typically lobulated, well-defined iso-/hypointense masses on T1, mainly homogeneous due to necrotic areas (Bednarova et al. 2018; Monteagudo et al. 2015) and hyperintense on T2. The LMS present a variable enhancement depending on the muscular and fibrous components present within the tumor, being the delayed enhancement major than the adjacent skeletal musculature (Fig. 1H). Hemorrhage and calcification are less common findings (Monteagudo et al. 2015). Due to their high cellularity and particularly in high-grade sarcomas, they have restrictions on DWI images (Bednarova et al. 2018). MR sequences showed the proper anatomy, especially when there are voluminous masses, making clear that the inferior cava vein was the origin of the tumors in our cases presented (Figs. 1, 2). Moreover, MR has the capacity to differentiate intraluminal mass from thrombus, a key feature that affects the prognosis (Ghose et al. 2018). Additionally, tumoral thrombosis typically enhances in MR sequences and expresses high SUVmax values, resembling the original tumor and differentiating from non-tumoral thrombosis.

**Fig. 4:**
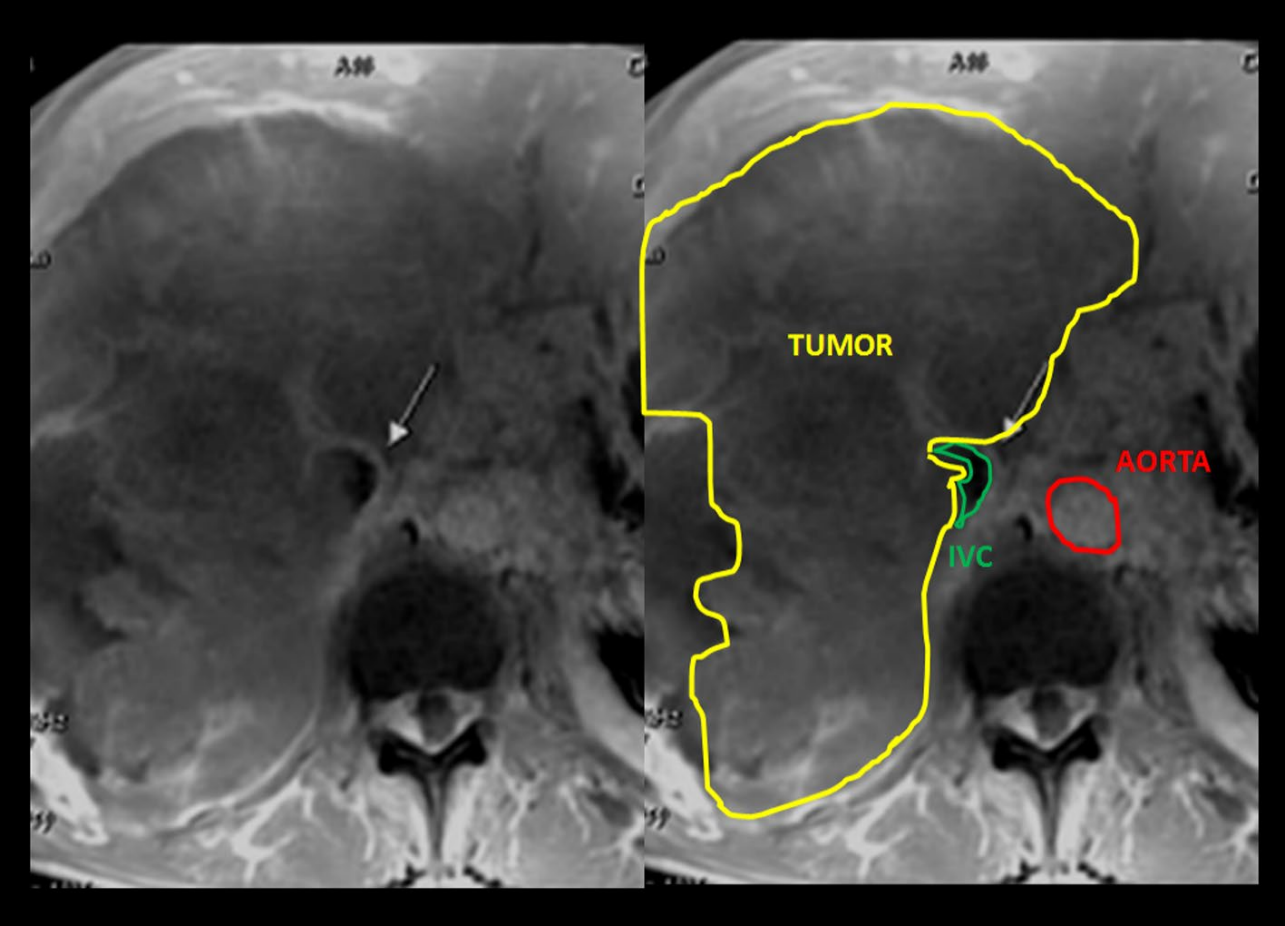
MR axial T1 enhancement scan and schematic representation shows the positive embedded sign seen in our second patient which compresses and displaces neighbor organs like right kidney and liver. This finding was key to locating the origin of the tumor

**Fig. 5:**
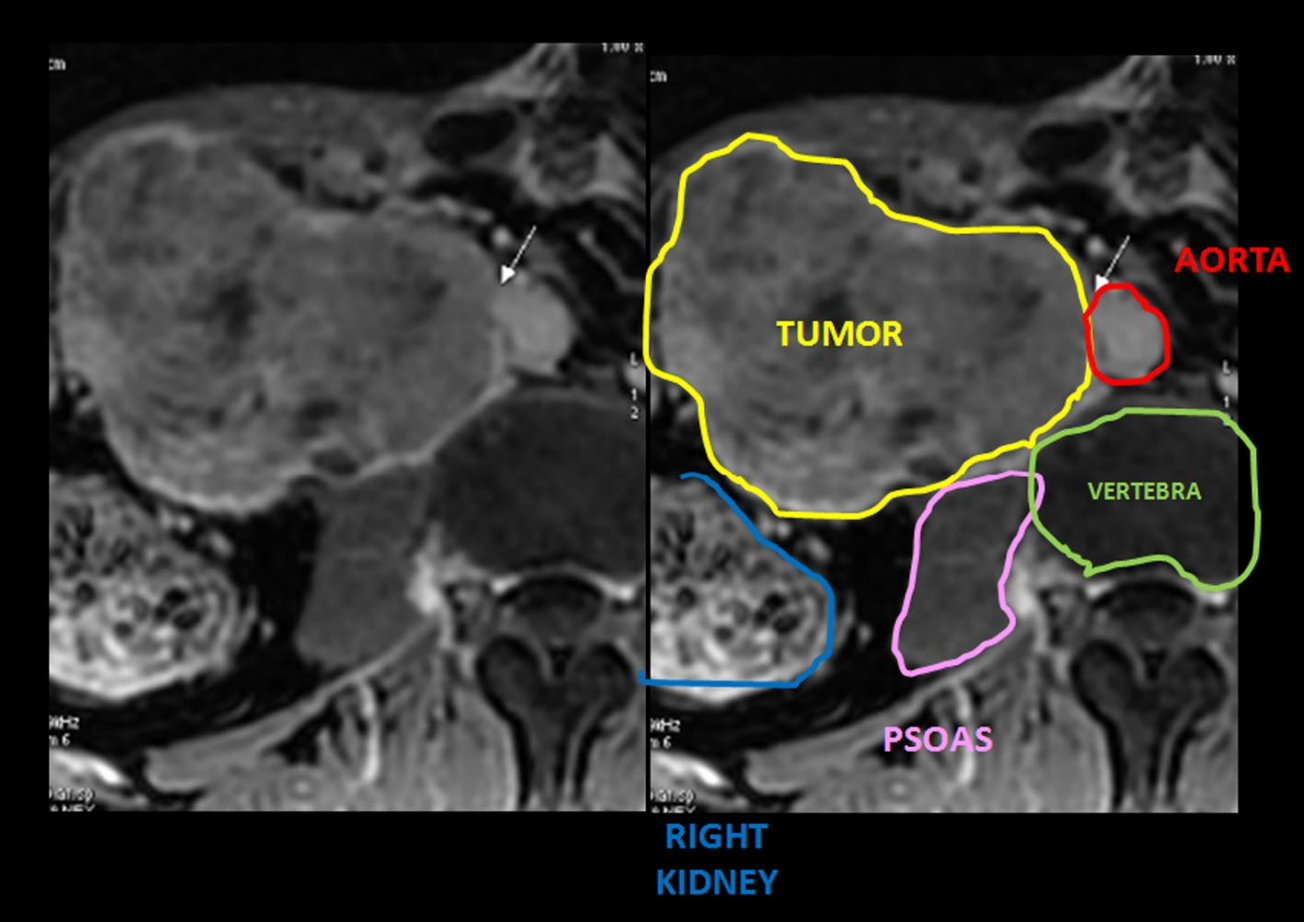
MR axial T2 scan from the first patient and schematic representation illustrates the negative peak sign from psoas muscle, dismissing its origin

Imaging signs are pivotal for both radiologists and nuclear medicine physicians; the main two are described. First and foremost, the “positive embedded organ” sign depicts a retroperitoneal mass which appears to be embedded in the tumor, meaning that it arises from retroperitoneal structures (Ghose et al. 2018; Nishino et al. 2003; Webb et al. 2013) (Fig. 4), while negative embedded sign accounts the opposite. Secondly, the “peak sign” illustrates that the edge of an organ is deformed like a peak shape, so it is likely that the mass arises from that organ; rounded edges implicate a negative peak sign and that the tumor compresses the organ, not rising there (Nishino et al. 2003) (Fig. 5).

PET imaging aims to identify metabolic activity as tumor uptake measured by the maximum tumor standard uptake value (SUVmax) is extremely valuable (Punt et al. 2009). This imaging grading measure for sarcomas is based on their histological behavior (Punt et al. 2009). Stephanie Punt et al. analyzed 39 patients with IVC LMS in which PET scan was previously performed for any treatment. They identified subjects aged 53 years in average, SUV max of 9.3 and tumor greatest dimension of 10 cm (mean values). Also, 48% of the histological analysis accounted for high-grade sarcomas. All these parameters concur with our patients' data. After a comprehensive analysis, they conclude that FDG PET is a forward and premature indicator of the tumor grade as a higher SUVmax correlates with both higher grade and maximum size (Punt et al. 2009). In conclusion, once again we reaffirm the position that the correlation between tumor grade and clinical impact relies on histological features (anticipated by PET images in a shortened period of time) as they may predict histological behavior of the malignant process having a direct impact on patients' prognosis and, hence, treatment.

Moreover, PET/MR not only examines the whole body but also allows, in a single diagnostic scan, to identify the presence of distant metastases (Fig. 2). This factor contributes to patients staging and prognosis. In light of the above, PET/MR has proved to have far more positive outcomes than other diagnostic techniques and the clue points are summarized in Table 2.

**Table 2:** Summary of teaching points

Teaching	Points
MR images	Pet scan
Peak sign and embedded organ sign	High SUVmax values
Hypovascular tumors with peripheral enhancement	Whole body scan
Hemorrhagic and calcification are unlikely	Time efficient
High DWI restriction	2 in 1 diagnosis study

## Conclusion

PET/MR is a noninvasive and low radiation exposure technique, not only used for characterizing leiomyosarcomas but also for staging and patient survival prediction. Consequently, the use of PET/MR may contribute to individualized patient treatment planning.

This hybrid methodology combines the advantages of morphofunctional examinations (MR) and the plus points of metabolic scan (PET), unified in a single examination, to provide a more accurate diagnostic approach.

We consider that the positive outcomes of this review will motivate the use of PET/MR in vascular sarcomas neoplasm in more centers worldwide, which may contribute to a deeper understanding of this rare neoplasm.

## Data Availability

The data supporting the conclusions of this article are included within the article.
